# Improved sensitivity of microperimetric outcomes for clinical studies in age-related macular degeneration

**DOI:** 10.1038/s41598-021-83716-w

**Published:** 2021-02-26

**Authors:** Yaniv Barkana, Susanne G. Pondorfer, Steffen Schmitz-Valckenberg, Hermann Russ, Robert P. Finger

**Affiliations:** 1 Galimedix Therapeutics, 3704 Calvend Lane, Kensington, MD 20895 USA; 2 The Glaucoma Innovation & Research Lab, The Sam Rothberg Glaucoma Center, Sheba Medical Center, Ramat Gan, Israel; 3 Department of Ophthalmology, University of Bonn, Bonn, Germany; 4 John A. Moran Eye Center, University of Utah, Salt Lake City, UT USA

**Keywords:** Outcomes research, Clinical trial design

## Abstract

To investigate sensitive outcome measures based exclusively on abnormal points in microperimetry testing of eyes with intermediate age-related macular degeneration (iAMD). 25 eyes of 25 subjects with iAMD had undergone 2 successive tests of mesopic microperimetry with the Macular Integrity Assessment Microperimeter (MAIA), using a custom grid of 33 points spanning the central 14 degrees of the macula. Each point was defined as abnormal if its threshold sensitivity was lower than 1.65 standard deviations (SD) (5%) or 2 SD (2.5%) than the expected threshold in healthy eyes according to the MAIA internal database. Among the 25 eyes there were 11.8 ± 9 and 8.4 ± 8.2 abnormal points at < 5% and < 2.5%, with mean deviation of thresholds from normal − 4.9 ± 1.2 dB and − 5.8 ± 1.5 dB, respectively. These deviations were greater, and their SD smaller, compared with the complete microperimetry grid, − 2.3 ± 2.0 dB. The 95% limits of agreement for average threshold between the 2 successive tests were smaller when only abnormal points were included. Analyzing only abnormal grid points yields an outcome parameter with a greater deviation from normal, a more homogenous dataset, and better test–retest variability, compared with analysis of all grid points. This parameter may thus be more sensitive to change, while moderately limiting the number of potential recruits. The proposed outcome measures should be further investigated as potential endpoints in clinical trials in iAMD.

## Introduction

Age-related macular degeneration (AMD) is the leading cause of blindness in the industrialized world, and remains an important unmet medical need^1^. Currently there is no approved treatment that can halt the progression of AMD from the early or intermediate (iAMD) forms to the advanced, visually-devastating forms. Clinical trials of novel interventions to stop or delay the progression of AMD are dependent on the use of sensitive outcomes to demonstrate efficacy. Accepted and validated functional endpoints are currently not available. The commonly used parameter of best corrected visual acuity is inadequate for trials of early or iAMD since it is affected only late in the disease spectrum, often only after progression to advanced AMD has occurred^2^.

Microperimetry using the Macular Integrity Assessment microperimeter (MAIA; CenterVue, Padova, Italy, now distributed by Icare, Finland) can be used to measure the threshold of differential light sensitivity in different locations in the macula, with the machine projecting the light stimulus directly on the retina while tracking the fundus using a scanning laser ophthalmoscope^3^. Such testing provides a wealth of data compared with the single-point assessment of foveal visual acuity or contrast sensitivity. Several reports have documented reduced microperimetry retinal sensitivity in eyes with iAMD despite preserved visual acuity^4–9^.

Some clinical trials in iAMD have a main goal of demonstrating improved vision, theorizing that removing the source of disease or providing “neuroenhancing” treatment may not only slow long-term neural degeneration, but also enhance function of sick retinal cells in the short-term, as has been suggested in a few early-phase clinical trials^10–12^. Of note, this concept of neuronal functional improvement is established in neurology, with several approved drugs in use that improve reduced function, e.g. cognitive improvement in Alzheimer or motor improvement in Parkinson’s disease^13^.

Investigators using MAIA have mostly used the parameter of mean sensitivity of all points in the testing grid to assess the degree of retinal function^4,5,7^. However, in eyes with iAMD, this parameter is depressed only slightly compared with normal eyes, and so may not be sensitive enough to show improvement with a therapeutic intervention. Moreover, a typical perimetric examination includes points that have “normal” sensitivity that is unlikely to improve in a clinical trial with any intervention. The inclusion of these points in the outcome measure may lead to “dilution” of the intervention’s effect on diseased points, thereby reducing the sensitivity of the outcome measure and ultimately potentially decreasing the chance of success of the clinical trial. On the other hand, it is conceivable that a parameter that is based on fewer points may be less repeatable, negating the advantages listed above. Thus, we investigated whether the sensitivity of microperimetric outcomes can be improved by using only those points that are defined as abnormal in the testing grid and thereby providing a potentially more sensitive outcome measure in clinical trials of visual function improvement in AMD.

## Methods

### Clinical data

This was a retrospective analysis of data obtained during a cross-sectional study at the Department of Ophthalmology, University of Bonn, Bonn, Germany, which has been reported previously^7^. The study was approved by the Institutional Review Board of the University Bonn. Written informed consent was obtained from all participants following an explanation of all tests involved. The protocol followed the tenets of the Declaration of Helsinki.

**Figure 1:**
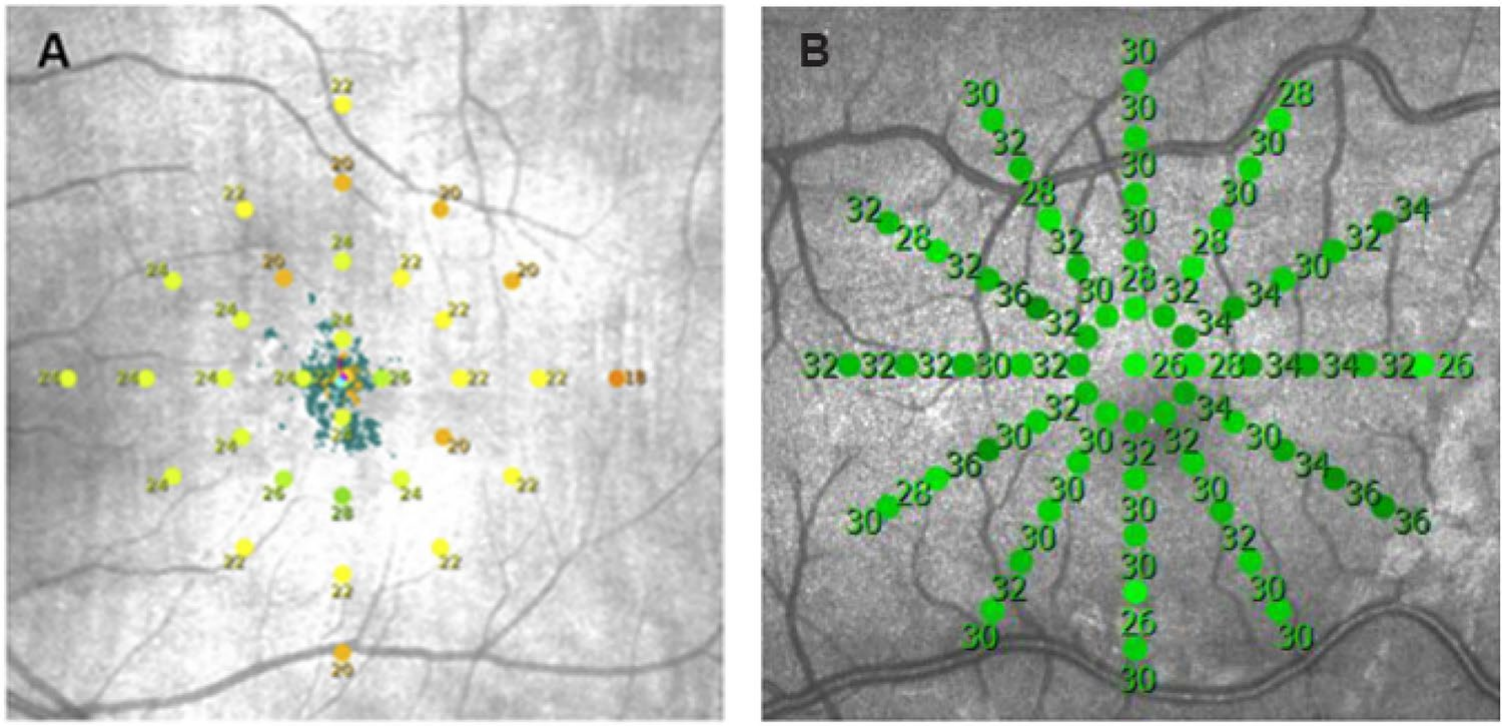
(**A**) Shows the layout of the study custom stimulus grid—33 points located at 0°, 1°, 3°, 5°, and 7° from fixation. (**B**) Shows (in larger magnification) the layout of the grid used by Centervue to create the Maia normative database—61 points located at 0°, 1°, 2°, 3°, 4°, 5° from fixation. It can be seen that the points overlap except at 7°.

Twenty-five eyes of 25 patients (age 67.7 ± 7.1) were included with iAMD defined as having drusen greater than 125 microns and/or any AMD pigmentary abnormalities without the presence of choroidal neovascularization (CNV) or geographic atrophy. In addition, 22 eyes of 22 controls in good retinal health (age 62.2 ± 4.4) were included. In the original study subjects had undergone two mesopic and two dark-adapted microperimetric examinations using the S-MAIA device with small breaks (maximum 5 min) between the examinations. In the present analysis only data from the mesopic exams were analyzed. A customized stimulus grid was used that consisted of 33 points located at 0°, 1°, 3°, 5°, and 7° from fixation (Fig. 1A). The rate of false-positive responses had to be less than 33%.

### Selection of testing grid points

We followed the rationale of looking at pointwise deviations from age-expected normal values rather than raw threshold sensitivity, as is customary in perimetric research in glaucoma. Ideally, for determining abnormality, a multi-center clinical study should employ a standard normative database, rather than each site separately defining a small normal population. Centervue, the manufacturer of MAIA, has incorporated an FDA-approved database in the machine’s software, derived from analysis of 494 healthy eyes in 270 subjects aged 21–86 (average 42.9), 17% of which were older than 60 years, using a 61-point grid covering the central ten degrees of the macula (i.e. test points up to a radius of five degrees eccentricity, MAIA operating manual, available at https://drive.google.com/file/d/1ldLyT7H0AADTsdYP1SyEpep1EbmChDsV/view, Fig. 1B). It calculates the expected normal threshold in dB = 32.3 − 0.06 * age, with the threshold being independent of location within the tested central 5 degrees; the standard deviation (SD) is 1.78 dB for all points, regardless of age or location (personal communication, Luca Zalunardo, Centervue Inc, USA). Against this background, the formula is also applicable to our data which include test points at seven degree excentricity from the fovea. We used this reference to calculate the amount of deviation from expected normal values in order to define individual perimetric points as abnormal. We observed that the average sensitivity of the healthy controls in the original study was 25.9 dB, which is 2.67 dB lower than predicted by the Maia normative formula cited above. Therefore, for the present analysis of deviation from normal, all measured sensitivity values in the iAMD database were adjusted by adding 2.67 dB. The adjusted mean grid threshold in the cohort’s healthy eyes was 28.5 ± 1.3 dB (range 25.6–30.8). In these eyes the SD of threshold values in each individual grid was, on average, 1.8 ± 0.4 (range 1.3–2.6). This figure is very similar to the SD of normal threshold at each point provided by the Maia’s database, which as noted above is 1.78 dB.

We defined each point in the grid as abnormal if its threshold was lower than 1.65 SD from the expected age-corrected normal threshold, i.e. 2.9 dB (1.78 dB × 1.65) lower. This means the measured value would be expected in less than 5% of points in healthy eyes, a commonly used clinical cutoff value when determining “normal” in perimetry^14^. In addition, we also used a more strict definition of abnormality as lower than 2 SD from expected value, i.e. 3.6 dB, expected in less than 2.5% of measurements in healthy eyes.

### Statistical analyses

Using descriptive statistics, we analyzed the potential use of these criteria in clinical trials of iAMD, if analysis of sensitivity is restricted to only those points in the grid that are abnormal. Specifically we described the number of abnormal points in each test, the mean sensitivity and deviation form presumed healthy thresholds of these points, and the number of eyes in the dataset that would qualify for a potential clinical trial requiring at least 5 abnormal points in a test. We also compared the repeatability of test results using only abnormal points vs. all grid points using Bland–Altman 95% limits of agreement^15^.

## Results

All threshold values are presented after adjustment as explained above.

In eyes with iAMD (first of 2 tests) mean grid threshold was 25.9 ± 2.1 dB (range 19.6–28.7 dB). The SD of threshold values within each grid was 2.4 ± 0.8 dB (1.4–4.2 dB). The deviation of mean total-grid threshold from the MAIA-predicted age-corrected value was − 2.3 dB ± 2.0 dB. This demonstrates that indeed macular threshold is decreased in eyes with iAMD in a non-homogenous way—in most tests threshold values span a range of roughly 10 dB, and there are many points with normal sensitivity.

**Figure 2:**
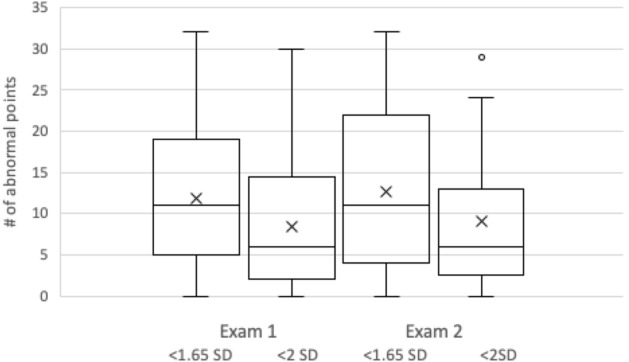
Boxplot showing the number of abnormal points in each microperimetry test in the study cohort of 25 eyes. The 2 leftmost plots represent the first exam, and the rightmost pair the second exam. In each pair, the left plot was generated when a point was defined as “abnormal” if its threshold sensitivity was lower than 1.65 SD (5%) of expected value in a healthy population; for the right plot abnormality was defined as < 2SD (2.5%) of normal. Each boxplot includes the maximum (upper whisker, excluding outliers), upper quartile (top of box), median (horizontal line in box), lower quartile (bottom of box), minimum (lower whisker, excluding outliers) and average (x) values. Outliers are represented by black dots.

Table 1 shows the number of abnormal points in the two successive mesopic exams using the two criteria of abnormality, together with the average threshold and deviation of these points. The number of abnormal points is also illustrated graphically in Fig. 2. The abnormal points were a minority, about a third (11.8 and 12.6 of 33) when we used the less strict criterion, and a fourth (8.4 and 9) when using the more strict criterion for abnormality. It is apparent that the number of abnormal points varies widely among eyes with iAMD. At the same time, the variability of the latter points was smaller as compared to the variability of all points. Specifically, the SD of the average sensitivity of all points in each exam was 2.0 dB, while it was smaller for abnormal points only, at 1.35 or 1.34 dB for the less strict criterion, and 1.6 or 1.8 dB for more strict criterion.

**Table 1:** Number of abnormal points in 2 successive mesopic Maia exams in 25 iAMD eyes using 2 criteria for abnormality, and the average threshold and deviation of these points.

	Exam 1			Exam 2		
	# of points (range,%)	Threshold (dB)	Deviation (dB)	# of points (range,%)	Threshold (dB) (*p* values vs Exam 1)	Deviation (dB)
Threshold lower than 1.65 SD (2.9 dB) of age-corrected normal	11.8 ± 9.12 (0–32) 38%	23.3 ± 1.35^1^	− 4.9 ± 1.2^1^	12.6 ± 9.6 (0–32) 40%	23.0 ± 1.34^2^ (*p* = 0.26)	− 5.2 ± 1.3^2^
Threshold lower than 2.0 SD (3.6 dB) of age-corrected normal	8.4 ± 8.2 (0–30) 27%	22.4 ± 1.8^3^	− 5.8 ± 1.5^3^	9 ± 8.9 (0–29) 29%	22.2 ± 1.6^4^ (*p* = 0.49)	− 6.0 ± 1.3^4^

^1^There was one eye with no abnormal points, so it was not included in this analysis.

^2^^–4^There were 2 eyes with no abnormal points, so they were not included in this analysis.

Regarding the mean deviation of abnormal points compared to age-correct normal values, it was significantly greater in the two exams, − 4.9 or − 5.2 dB for the less strict criterion of abnormality and − 5.8 or − 6.0 dB for the more strict criterion, versus only − 2.3 dB for the total grid. The SD of the deviations was even smaller than the SD of the mean threshold sensitivities and ranged between 1.2 and 1.5 dB.

We computed Cohen’s d values (https://lbecker.uccs.edu/) for the average deviation of abnormal points, in both exams and according to both criteria of abnormality, with respect to the deviations in the 44 healthy eyes. The four values ranged between 3.94 and 4.5, indicating very high specificity.

To provide a clinically meaningful analysis, an outcome measure needs to be based on a certain minimal number of data points. Here we chose an arbitrary cutoff of 5 abnormal points that should be present in the exam. There were 19 eyes (76%) that matched this criterion using the less strict criterion of abnormality in both exams, and 14 (56%) or 16 (64%) with the more strict criterion, as shown in Table 2. These numbers decreased slightly if an eye had to match the criterion in both the first and second examinations.

**Table 2:** The number of iAMD eyes out of the cohort of 25 eyes with at least 5 abnormal points using 2 criteria for abnormality, and the average threshold and deviation of these points.

	Exam 1			Exam 2			Both exams		
	# eyes (%)	Threshold (dB)	Deviation (dB)	# eyes (%)	Threshold (dB)	Deviation (dB)	# eyes (%)	Threshold (dB)	Deviation (dB)
Threshold lower than 1.65 SD	19 (76%)	23.1 ± 1.4	− 5.1 ± 1.2	19 (76%)	23.0 ± 1.4	− 5.2 ± 1.3	18 (72%)	23.0 ± 1.4	− 5.2 ± 1.2
Threshold lower than 2.0 SD	14 (56%)	22.2 ± 1.8	− 6.0 ± 1.4	16 (64%)	22.2 ± 1.8	− 6.1 ± 1.6	13 (52%)	22.3 ± 1.8	− 5.9 ± 1.5

**Figure 3:**
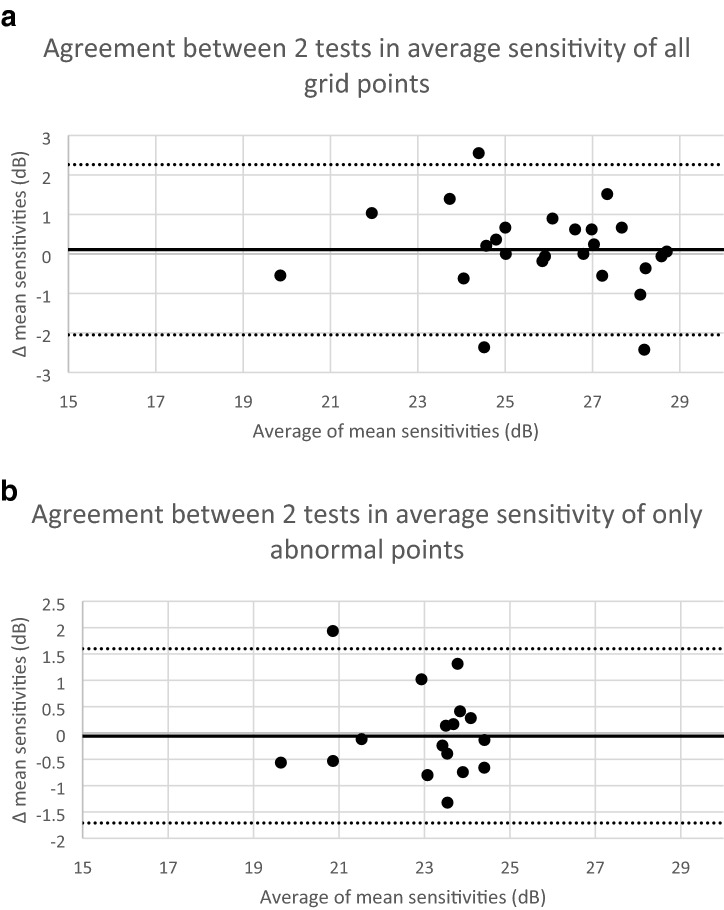
Bland–Altman plots for the average sensitivity threshold in 2 successive mesopic microperimetry tests when all grid points were included (**a**, complete cohort of 25 eyes) and when only abnormal points at < 1.65 SD (5%) were included (**b**, 18 eyes with at least 5 such points). The x-axis shows the mean of average sensitivity for each pair of repeated tests for each subject, the y-axis the difference between the average sensitivity in the two tests (first test–second test). The overall mean difference is represented by the central bold line, and the 95% Limits of agreement are marked by the upper and lower dashed lines.

Table 1 and Fig. 2 demonstrate that results were very similar between the two repeated tests. For this pair of tests in eyes with iAMD, the 95% limits of agreement for average threshold are shown in Fig. 3. These were 4.2 dB when the total grid was included (Fig. 3a, n = 25 eyes). When the average threshold of only abnormal points (at < 1.65 SD) in eyes with at least 5 such points in both exams was compared between the two tests, the 95% limits of agreement were smaller at 3.2 dB (Fig. 3b, n = 18 eyes), implying better repeatability.

**Figure 4:**
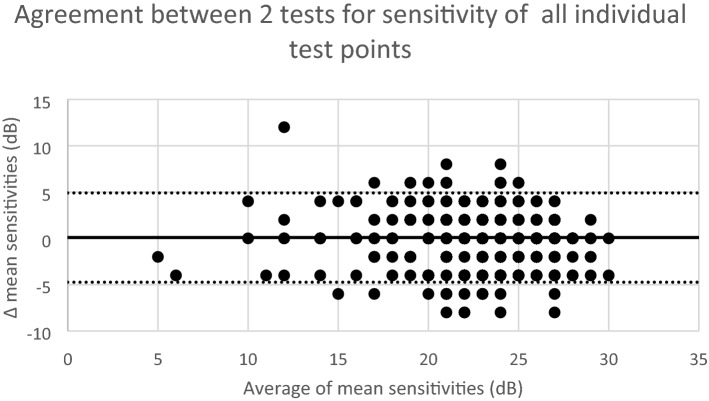
Bland–Altman plots for the sensitivity threshold of all individual tested grid points in 2 successive mesopic microperimetry tests when all grid points were included for the complete cohort. The x-axis shows the mean sensitivity for each point in the pair of repeated tests for each subject, the y-axis the difference between the average sensitivity in the two tests. The overall mean difference is represented by the central bold line, and the 95% limits of agreement are marked by the upper and lower dashed lines.

Figure 4 shows the agreement between all individual tested points. Clearly, 95% limits of agreement here are much wider compared with those of the averages above, spanning 9.6 dB.

## Discussion

The presented analysis highlights a number of potential advantages in using an outcome measure that is derived from abnormal points only rather than all the points of the MAIA microperimetry grid in eyes with iAMD. First, assuming that our cohort is typical of a population with iAMD, the abnormal points represent a minority in relation to all test points. Thus, using the average sensitivity of all grid points would clearly “dilute” an intervention’s effect on diseased retinal foci, thereby reducing the chance to observe a significant effect and thus the chance for a successful clinical trial. Second, there was a large variability of average total-grid threshold values among this cohort of eyes with iAMD that was reduced when the average threshold of only the abnormal points was considered; the variability was reduced further when the average deviation rather than the average threshold was considered. Third, the average deviation of abnormal points is, as expected, significantly larger than the average deviation of the total grid. Thus, by providing a study sample which is more homogenous and also has a greater initial deviation, these results suggest that based on the proposed approach of selecting only abnormal testing points for further analysis, sensitivity for change in outcome measure can be improved and future studies in iAMD may require smaller sample sizes and/or shorter follow-up.

As clinical studies involve repeated testing, better repeatability of the outcome measure is also helpful in reducing required sample size. The Bland–Altman analysis of the repeatability of measurements within the same subject showed a lower 95% LoA when only abnormal points were used. This is reassuring since averaging fewer points in the outcome measure may be theorized to result in more “noise” due to the inherent test–retest variability of subjective perimetry testing, especially in elderly subjects. This theory did indeed materialize when we tested the repeatability of pointwise (individual points) thresholds, as the variability was significantly higher compared with both averaged sensitivity of all or only abnormal points.

In the original report, the deviation of iAMD values from the average (not age-corrected) threshold in the study’s own control population was − 2.6 ± 2.1 dB which is very similar to the adjusted deviation of the MAIA’s internal normative database in this analysis (− 2.3 ± 2.0 dB)^7^. Considering that the study’s control population was slightly younger than the iAMD cohort, and so would be expected to have a slightly lower threshold had it been perfectly age-matched with subjects with iAMD, our observed data lend support to the validity of using the MAIA’s internal database. Moreover, these values are similar to average deviations in eyes with iAMD reported by others. Cocce et al. compared 47 eyes with iAMD defined as “AMD with the presence of drusen larger than 63 um and pigmentary anomalies” (age 70.4 ± 6.9) with 21 healthy controls (age 71.7 ± 7.4) using a standard grid of 37 points within a 10 degree diameter, and reported a mean deviation of − 1.8 dB between the groups^4^. The inclusion of eyes with smaller drusen, i.e. less severe disease, might partly explain the lower average deviation compared with our findings. Roh et al. compared 71 eyes with iAMD according to AREDS2 classification (age 69.7 ± 5.3) with 46 control eyes (age 65.4 ± 6.6.) using the same grid, with reported average deviation of − 2.7 dB between the groups^5^.

Limiting study enrollment to eyes with a defined degree of dysfunction means that some potential subjects would be excluded, potentially complicating enrollment. In our cohort, 18 of 25 eyes met the criterion of at least 5 abnormal points in both tests, leading to 28% of potential study subjects being excluded. Targeting clinically more advanced iAMD cases for enrolment might limit the amount of screen failures. For example, it has been shown that dark adaptation, another functional outcome measure in eyes with iAMD, was reported to be progressively worse when the fellow eye also had large drusen or advanced AMD^16^. As another example, using MAIA to assess the 5 central points (central 1 degree) in a macular test, eyes with both drusen > 125 microns and pigmentary abnormalities had a lower sensitivity threshold compared with eyes with drusen only^6^. Thus, retinal morphology might aid considerably in pre-selecting iAMD patients and reduce the number of screen failures on subsequent microperimetry testing.

So called normative databases integrated into functional testing devices by manufacturers may or may not reflect the actual “true normal”. Due to the discrepancy identified in our study we reviewed other published studies which used a MAIA device and reported average thresholds in healthy eyes. We identified three such studies and calculated the predicted value according to the MAIA’s normative database by using the reported mean age of the healthy population. Based on this, the reported mean threshold sensitivity was within 1 dB of the calculated prediction for all studies, with − 1.02 dB^4^, + 0.9 dB^6^ (average sensitivity 29.3 dB; personal communication Zhichao Wu), and + 0.3 dB^5^. This highlights that the database integrated in the MAIA device can be used albeit requires a certain caution and re-evaluation in each sample.

These results are based on repeated tests done within the same visit. Further study is needed to verify that our results are observed when inter-visit tests are compared. We note that while analyzing only abnormal points may increase endpoint sensitivity when seeking improved function or slowing deterioration in areas of retina that are dysfunctional at baseline, this methodology does not allow the detection of changes in areas that are normal at baseline. Therefore this approach might underestimate any effect related to slowing of progression rather than improving function by any tested intervention.

In conclusion, we propose a framework for the construction of an outcome measure for clinical trials of visual function improvement in iAMD that consists only of microperimetry points that are abnormal at baseline. Despite limiting the analysis to a particular subset of points, most subjects in this iAMD cohort could be enrolled. Advantages include a greater average deviation allowing more ample opportunity for observable effect of any intervention, a more homogenous dataset, and excellent test–retest variability.
